# The structure of the bacterial iron–catecholate transporter Fiu suggests that it imports substrates via a two-step mechanism

**DOI:** 10.1074/jbc.RA119.011018

**Published:** 2019-11-11

**Authors:** Rhys Grinter, Trevor Lithgow

**Affiliations:** ‡ School of Biological Sciences, Monash University, Clayton, 3800 Victoria, Australia; § Infection and Immunity Program, Biomedicine Discovery Institute and Department of Microbiology, Monash University, Clayton, 3800 Victoria, Australia

**Keywords:** membrane transport, X-ray crystallography, Escherichia coli (E. coli), protein structure, outer membrane, siderophore, bacterial outer membrane, ferric iron uptake (Fiu), iron acquisition, solute uptake, TonB-dependent transporter

## Abstract

The ferric iron uptake (Fiu) transporter from *Escherichia coli* functions in the transport of iron–catecholate complexes across the bacterial outer membrane, providing the bacterium with iron, which is essential for growth. Recently it has become clear that Fiu also represents a liability for *E. coli* because its activity allows import of antimicrobial compounds that mimic catecholate. This inadvertent import suggests the potential utility of antimicrobial catechol siderophore mimetics in managing bacterial infections. However, to fully exploit these compounds, a detailed understanding of the mechanism of transport through Fiu and related transporters is required. To address this question, we determined the crystal structure of Fiu at 2.1–2.9 Å and analyzed its function in *E. coli*. Through analysis of the Fiuo crystal structure, in combination with *in silico* docking and mutagenesis, we provide insight into how Fiu and related transporters bind catecholate in a surface-exposed cavity. Moreover, through determination of the structure of Fiu in multiple crystal states, we revealed the presence of a large, selectively gated cavity in the interior of this transporter. This chamber is large enough to accommodate the Fiu substrate and may allow import of substrates via a two-step mechanism. This would avoid channel formation through the transporter and inadvertent import of toxic molecules. As Fiu and its homologs are the targets of substrate-mimicking antibiotics, these results may assist in the development of these compounds.

## Introduction

The outer membrane of Gram-negative bacteria provides a selective permeability barrier to molecules with a molecular mass greater than ∼600 Da (1). The barrier provides superb protection against antimicrobials and toxic compounds (2). The selective permeability of the outer membrane also restricts the uptake of nutrients, including iron, which, although abundant on Earth, is often growth-limiting because of its insolubility under the oxidizing conditions of the terrestrial atmosphere (3, 4). Aerobic organisms solubilize iron through the formation of iron-chelating chemicals (siderophores) or incorporate it into other organic structures, such as the porphyrin ring of heme or iron-binding proteins (5–7). These iron-containing complexes are larger than the diffusion limit of the bacterial outer membrane, and so, to obtain the iron required for growth, bacteria have evolved transporters capable of selectively binding and importing iron-containing complexes (8, 9). Members of the TonB-dependent transporter (TBDT)^3^ family drive transport of their substrates through interaction with the energy-transducing protein TonB (10). TBDTs are highly divergent in sequence but share a common structural architecture, consisting of a 22-stranded transmembrane β-barrel, the lumen of which is selectively occluded by a globular plug domain (8).

The ability of TBDTs to import large substrates comes at a cos, an evolutionary arms race exists between TBDT-producing bacteria and organisms seeking to kill them by hijacking these transporters (11–15). Both small-molecule and protein antibiotics mimic TBDT substrates, leading to their inadvertent import into the bacterial cell (12, 16–21). Catecholates are one of the four recognized classes of siderophores (22); they have a strong affinity for iron and are abundant secretion products of both bacteria and fungi (23, 24). The ferric iron uptake (Fiu) transporter is a TBDT responsible for import of molecules containing the catecholate functional group (2, 25). In addition to its role in iron uptake, Fiu has also been shown to be important for sensitivity to antimicrobials that share the common feature of a catecholate functional group or the analogous dihydroxypyridine moiety (21, 25, 26). It is thought that the presence of these chemical mimetics leads to their inadvertent import into the bacterial cell via Fiu (27). This Fiu-mediated sensitivity is observed even in the absence of iron because Fiu functions in import of catecholate-containing molecules, seemingly independent of their size or Fe coordination state (18). The potential of antimicrobial catechol siderophore mimetics as therapeutic agents is demonstrated by development of the 3,4-dihydroxypyridine–containing sulbactam BAL30072 by Basilea Pharmaceutica and the catecholate-containing cephalosporin cefiderocol by Shionogi Inc. BAL30072 and cefiderocol have entered clinical trials for treatment of infections by Gram-negative bacteria in the human lung and urinary tract, respectively (28, 29).

In this study, we show that, although Fiu and its homologs PiuA and PiuD function in import of catecholate siderophores (25, 30), they are evolutionarily distinct from the well-studied catecholate siderophore transporters FepA/PfeA and Cir. To investigate the substrate import mechanism of the Fiu/Piu TBDT subgroup, we solved the crystal structure of Fiu. Analysis of this structure in combination with *in silico* docking and mutagenesis identified an external substrate-binding site in Fiu, which is conserved among diverse TBDTs. In addition, the presence of a large selectively gated internal chamber in Fiu, capable of accommodating a Fe–siderophore complex, suggests that these transporters may function via a two-step gating mechanism.

## Results

### Fiu is a member of a distinct clade of iron–catecholate transporters

**Figure 1.**
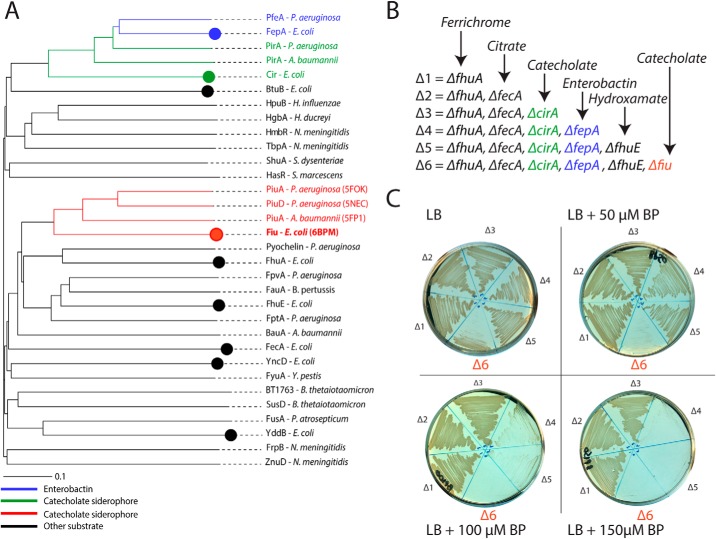
**Fiu belongs to a distinct group of catecholate siderophore transporters.** *A*, a phylogenetic tree of diverse, functionally characterized TBDTs, showing that Fiu forms a clade with PiuA/D that is distant from the catecholate siderophore transporters FepA and Cir. Catecholate-transporting TBDT subgroups are color coded: *blue*, enterobactin-transporting; *green*, nonenterobactin-transporting FepA-related; *red*, nonenterobactin-transporting Fiu-related. *Circles* represent TBDTs present in *E. coli* BW25113. *B*, a scheme showing the sequential deletion of TBDTs in *E. coli* BW25113 utilized in this study, colored as in *A. C*, strains from *B* grown on LB agar in the presence of 0–150 μm 2,2′-bipyridine (*BP*). Sequential loss of FepA and Fiu leads to defects in the ability of strains to grow under iron-limiting conditions.

It has been demonstrated previously that the archetypical *Escherichia coli* strain BW25113 possesses three TBDT transporters that function in the uptake of catecholate siderophores: FepA, Cir, and Fiu (25). FepA imports the endogenously produced siderophore enterobactin with high affinity (31), whereas Cir and Fiu have been shown to transport monomeric catecholate compounds, either alone or in complex with iron (32). Although these transporters recognize a common functional group, they share limited amino acid sequence identity, and their evolutionary relationship remained undetermined. To resolve this question, we performed phylogenetic analysis of these transporters in the context of a panel of diverse TBDTs of known structure and/or function. This analysis revealed that, although Cir and FepA belong to the same clade of the TBDT phylogram, Fiu belongs to a distal clade with the TBDTs PiuA and PiuD that also mediate catecholate transport (30, 33) (Fig. 1*A*). A wider analysis of this Fiu/Piu clade, identified by HMMER search revealed that related sequences are widespread in proteobacteria (Fig. S1 and Tables S1 and S2). Based on clustering analysis of these sequences, all members of this expanded group are more closely related to Fiu than to either Cir or FepA (Fig. S1). Furthermore, consistent with our phylogram, these transporters are more closely related to the hydroxamate–siderophore transporter FhuA than either Cir or FepA (Fig. 1*A* and Fig. S1). These data demonstrate that, although Fiu, Cir, and FepA all transport catecholate-containing substrates, Fiu is evolutionarily distinct from Cir and FepA and may have arrived at its substrate specificity because of convergent evolution between these transporters.

TBDT mediated iron uptake-systems are generally redundant to provide the means to obtain iron under a variety of environmental conditions (23, 34, 35). Thus, to dissect the specific role of Fiu, each of the six TBDTs known to be involved in iron acquisition were sequentially deleted from *E. coli* BW25113 in the following order: Δ*fhuA* (ferrichrome transporter), Δ*fecA* (ferric citrate transporter), Δ*cirA* (Fe–catecholate siderophore transporter), Δ*fepA* (enterobactin transporter), Δ*fhuE* (rhodotorulic acid transporter), and Δ*fiu* (Fig. 1*B*). The phenotypes of these mutants were assessed by growth on LB agar containing the iron chelator 2,2′-bipyridine (Fig. 1*C*). The first three receptors (FhuA, FecA, and CirA) were dispensable for growth in this assay, but subsequent loss of the enterobactin receptor FepA affected the growth of the mutant strain at 2,2′-bipyridine concentrations of more than 50 μm (Fig. 1*C*). There was no further phenotype from loss of the coprogen receptor FhuE under our assay conditions (Δ5, Fig. 1*C*). Subsequent loss of Fiu led to impaired growth on LB agar and completely prevented growth at 2,2′-bipyridine concentrations of 50 μm or higher (Δ6, Fig. 1*C*). This growth defect was restored either by in trans complementation with a plasmid encoding Fiu (Fig. S2) or supplementation of the growth medium with Fe(II)SO_4_.

These data show that Fiu is able to provide iron to the cell when present as the sole outer-membrane iron transporter. As Fiu is unable to transport endogenously produced enterobactin (31, 36), in this context, it most likely functions to transport enterobactin breakdown products (*i.e.* 2,3-dihydroxybenzoyl-l-serine (DHBS)) in complex with iron. The inability of Fiu to support growth in the presence of high concentrations of 2,2′-bipyridine may be due to the lower affinity of the monomeric catecholates for Fe^3+^ or a low affinity of Fiu for the Fe–DHBS complex.

### The crystal structures of Fiu reveal a large, gated internal chamber

**Figure 2.**
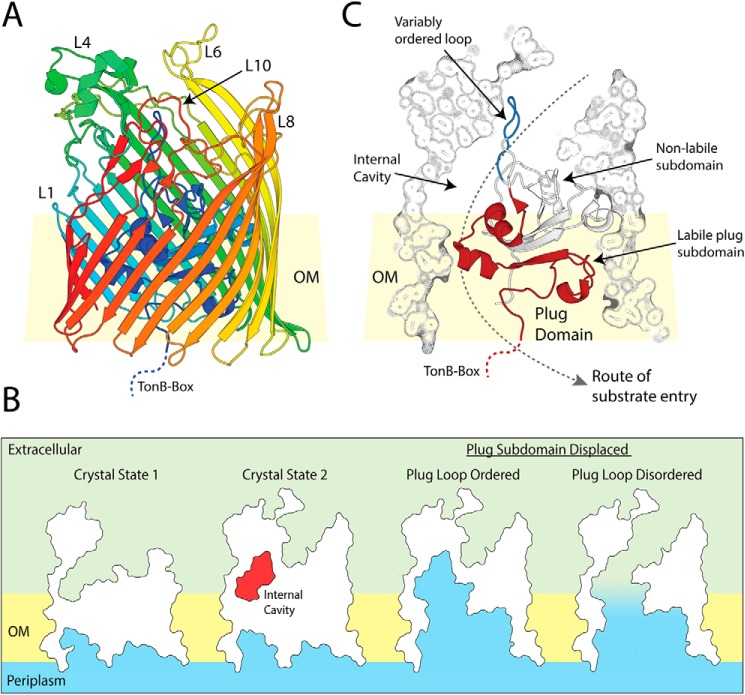
**The crystal structure of Fiu reveals a large gated internal cavity.** *A*, the crystal structure of fully ordered Fiu (crystal state 2), shown as a cartoon representation with *rainbow colors* running from N-terminal (*blue*) to C-terminal (*red*). *B*, cutaway outline representation of Fiu crystal structures, showing the internal cavity selectively occluded in crystal state 2 as well as the effect of removal of the labile subdomain of the TBDT plug on Fiu channel formation through the membrane. *C*, composite cutaway view of Fiu, showing the N-terminal plug domain as a cartoon, with the variably ordered plug loop (*blue*) and labile plug subdomain (*red*) highlighted. Outer membrane is abbreviated to OM in this figure.

**Figure 3.**
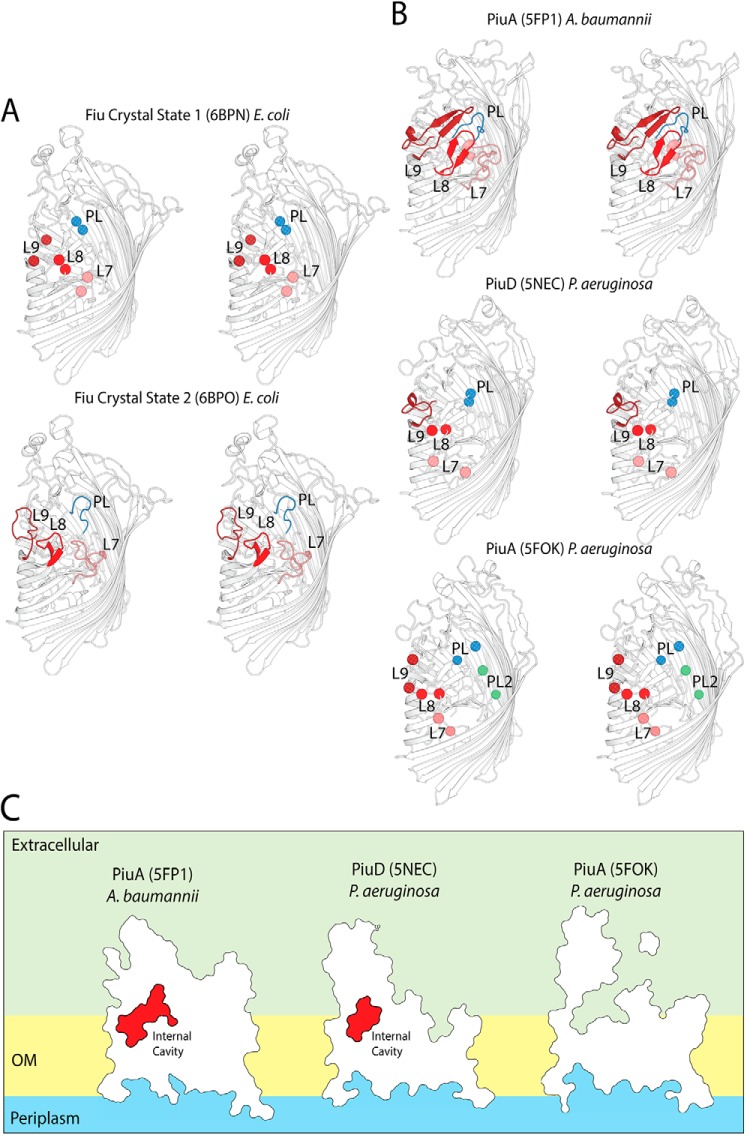
**Extracellular loop stability in crystal states of Fiu and PiuA/PiuD.** *A*, stereo cartoon view of Fiu in crystal states 1 and 2, illustrating the variably ordered extracellular barrel loops 7, 8, and 9 (*L7–L9*) and the plug domain loop (*PL*). Loops are color-coded as follows: L7, *salmon*; L8, *red*; L9, *brick red*; plug domain loop, *blue*. Loop termini are shown as *spheres* where the loop is disordered. *B*, PiuA and PiuD crystal structures from *P. aeruginosa* and *A. baumannii* presented as for Fiu in *A*. The crystal structures of PiuA and PiuD display an analogous pattern of loop order/disorder to that observed for Fiu. *C*, cutaway outline representation of PiuA and PiuD, showing the presence of a selectively gated internal cavity. Outer membrane is abbreviated to OM in this figure.

To obtain insight into the structural basis of substrate binding and import by Fiu, we determined the structure of Fiu by X-ray crystallography (Table S3). The structure of Fiu consists of a 22-stranded transmembrane β-barrel characteristic of the TBDT superfamily, with a number of extended extracellular loops that might serve in the initial steps of substrate binding (Fig. 2*A*). In agreement with our phylogenetic analysis (Fig. 1*A*), the DALI web server (37) identified PiuA from *Acinetobacter baumannii* (PDB code 5FP1) as the closest structural homolog to Fiu in the PDB (Dali server Z-score = 45, backbone atom RMSD of 6.182 Å, 33% amino acid identity). The structure of Fiu was solved in three different crystal forms, revealing Fiu in two distinct states (Table S3). In crystal state 1, extracellular loops 7–9 of the β-barrel were disordered, as was the extended extracellular loop of the N-terminal plug domain, which occluded the lumen of the Fiu β-barrel (Fig. 3*A*). In contrast, in crystal state 2, the entire polypeptide chain (amino acids 50–760) C-terminal of the TonB box (which is disordered in both crystal forms) could be modeled into the available electron density (Figs. 2*A* and 3*A*). The disorder of the plug domain loop in crystal state 1 opens a large cavity in the interior of Fiu to the external environment, whereas, in crystal state 2, this cavity is present but occluded in the lumen of the Fiu barrel (Fig. 2*B*). Interestingly, analysis of the structures of PiuA from *A. baumannii* and PiuA and PiuD from *Pseudomonas aeruginosa* revealed crystal states analogous to Fiu (Fig. 3, *B* and *C*) (30, 33). In PiuA from *A. baumannii,* all extracellular loops are ordered, with the plug loop occluding the entrance to an internal cavity (Fig. 3, *B* and *C*). In PiuA and PiuD from *P. aeruginosa*, the extracellular and plug loops are disordered, with the external cavity of PiuA exposed to the external environment (Fig. 3, *B* and *C*).

Utilizing force spectroscopy, Hickman *et al.* (37) showed that the N-terminal plug domain of TBDTs consists of labile and nonlabile subdomains. Upon substrate binding, TonB is recruited to the TonB box at the N terminus of the TBDT and facilitates reversible displacement of the labile subdomain of the TBDT plug via mechanical energy provided by the proton-motive force (37, 38). In support of this study, it has been shown that a number of charged residues at the interface between the labile and nonlabile subdomains in TBDTs are important for substrate transport but not binding (39). Based on these studies, we identified that the labile subdomain of the Fiu plug extends from the N terminus of the protein to the start of the extracellular plug domain loop, which is selectively ordered in our crystal structures (Fig. 2*C*). In crystal state 2, removal of the labile subdomain opens the internal cavity of Fiu to the periplasm, but because of the presence of the plug loop, this does not create a membrane-spanning channel. In crystal state 1, removal of this subdomain opens a large channel between the periplasm and the external environment (Fig. 2*B*).

TBDTs selectively transport their substrate across the outer membrane while preventing antibiotics and other deleterious molecules from entering the cell (8). Therefore, it is likely to be undesirable for Fiu to exist in the open-channel state, which would result from simultaneous displacement of the plug subdomain and disorder of the plug domain loop. The internal cavity we observed in our structure, gated by the selectively ordered plug loop, may provide a solution to this problem. The internal cavity is large enough (∼3200 Å^3^) to accommodate a Fe–siderophore complex. If a siderophore entered this chamber prior to removal of the labile plug subdomain, then it could enter the periplasm without formation of a membrane-spanning channel through the pore of Fiu.

### In silico docking suggests that Fiu possesses multiple substrate-binding sites

To determine the substrate-binding site of Fiu, we attempted cocrystallization and soaking of Fiu crystals in the presence of the monomeric catecholate compounds DHBS and 2,3-dihydroxybenzoic acid (DHB) in complex with Fe^3+^. These monomeric catecholates form a 3:1 complex with a single Fe^3+^ ion at the center. Despite the presence of DHBS at a high concentration (100–1000 μm) during crystallization screening and soaking and the resulting Fiu crystals diffracting well (2.8–2.0 Å), no electron density corresponding to DHBS was observed in the resulting density maps. Some crystals of Fiu grown in the presence of high concentrations of Fe–DHB (1 mm) exhibited the characteristic purple color of the Fe–catecholate complex (Fig. S3*A*). Although these crystals only diffracted to low resolution (anisotropic diffraction, 3.2–5.9 Å) (Table S4) Fo-Fc densities attributable to two Fe–DHB complexes were observed (Fig. S3*B* and Data S1). However, these Fe–DHB complexes were located on the side of the Fiu barrel, distal from the extracellular binding pocket, and were involved in crystal packing, suggesting that they are bound nonspecifically (Fig. S3*C*). Although it has been demonstrated previously that Fiu is capable of transporting DHB and DHBS *in vivo* (32), our inability to obtain a legitimate cocrystal structure suggests that Fiu has a low affinity for these compounds. As TBDTs generally bind their ligands with very high affinity (8), this suggests that monomeric catecholate compounds may not be the preferred substrate for Fiu, and its target siderophore remains unidentified.

**Figure 4.**
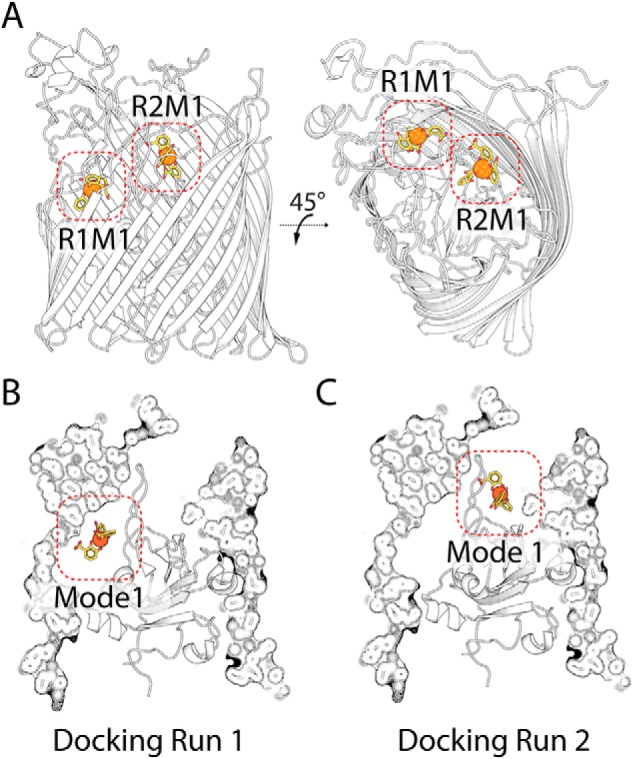
**Top-ranked docking solutions between Fiu and the Fe–DHB substrate.** *A*, the location of the top-ranked docking modes in a cartoon representation of Fiu, for docking run 1 (*R1M1*), which includes the entire extracellular portion of the transporter, and docking run 2 (*R2M1*), in which the internal cavity is excluded from the search area. *B*, the same docking solutions as in *A*, with a cutaway composite view of Fiu.

To determine potential substrate-binding sites in Fiu, we performed *in silico* docking between Fiu and Fe–DHB using Autodock Vina (40). The rationale for this experiment is that, although Fe–DHB appears to be a low-affinity ligand, the ability of Fiu to transport it suggests that the high-affinity substrate for this transporter is likely to be a catecholate siderophore, which would contain a Fe^3+^–catecholate complex analogous to DHB. Thus, although the results should be interpreted cautiously, docking with Fe–DHB provides an indication of substrate-binding sites in Fiu. Two docking runs were performed (Data S1). For the first run, the entire extracellular portion of closed Fiu (crystal state 2) was defined as the search area. In this experiment, the majority of the solutions placed Fe–DHB in the internal chamber of Fiu, with the third most favored solution positioning Fe–DHB in the extracellular cavity of the protein (Fig. 4, *A* and *B*, and Fig. S4 and Table S5). Although the internal cavity would be inaccessible to Fe–DHB in the closed state, because of the fully ordered plug loop, it would be accessible in state 1, as this loop is disordered. In the second docking experiment, the internal cavity was excluded from the search area. In this experiment, all solutions placed Fe–DHB in the Fiu extracellular cavity, with the majority of solutions clustered at a single location (Fig. 4, *A* and *C*, and Fig. S4). Suggestively, the top-ranking solution from this docking run was identical to the third top solution from the first experiment. Taken together, these results suggest that Fiu possesses a binding site capable of accommodating a Fe–catecholate complex in its extracellular cavity. In addition, these data show that the internal cavity of Fiu is capable of accommodating the Fe–DHB complex.

### The location of the putative external Fiu substrate-binding site is conserved among TBDTs

**Figure 5.**
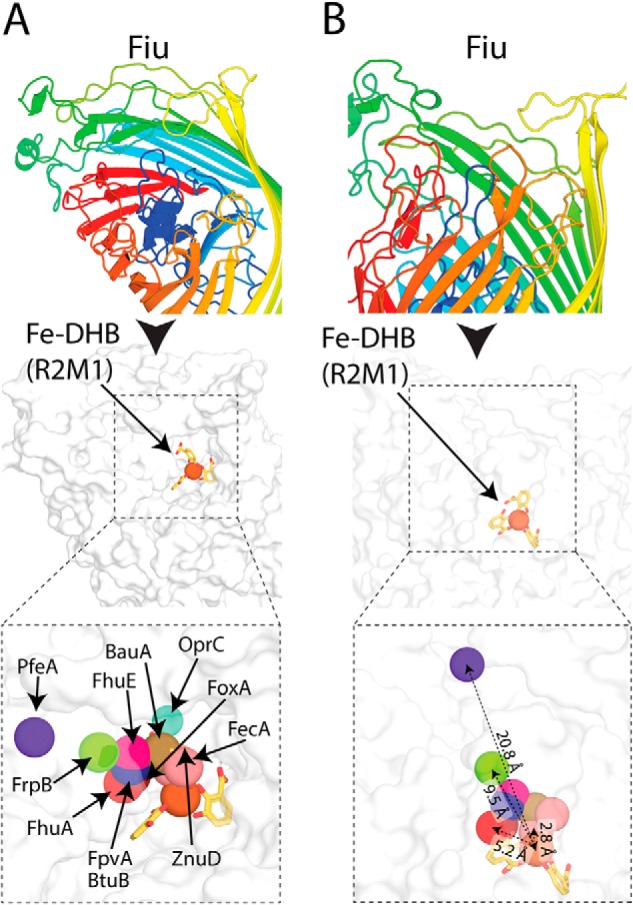
**The location of the Fiu putative external Fe–DHB binding site compared with other TBDT substrate complexes.** *A*, the location of the Fe–DHB complex docked with Fiu compared with that of other substrates bound to in the crystal structures of superimposed TBDTs. Fiu is shown as a *cartoon rainbow* and in the same view as a *white surface representation* below. The location of the metal centers of different TBDT substrates are shown as *colored spheres* and labeled. *B*, the Fiu Fe-DHB docked complex shown as in *A* but in a different orientation. Representative distances between Fe–DHB and the TBDT substrate metal ions are shown, colored as in *A*.

To validate the external substrate-binding site identified in our docking analysis, we compared its location with the substrates of other TBDTs that have been structurally characterized. For this analysis, we selected 12 nonredundant TBDT–ligand structures (Table S6) (38, 41–49) and superimposed them with the structure of Fiu. In 11 of these 12 structures, the substrate bound in an analogous location to our Fe–DHB docking solution, with the metal ion of the respective ligands located between 2.8 and 9.5 Å from the Fe of Fe–DHB in our docked complex (Fig. 5). The 12th structure, the whose ligand did not colocalize with Fe–DHB, is the enterobactin transporter PfeA from *P. aeruginosa* (Fig. 5). In this structure, the binding of enterobactin in PfeA occurs on the external face of the extracellular loops of the transporter, which entirely encloses the entrance to the lumen of the barrel (42). It has been demonstrated that PfeA binds enterobactin via two-site binding and that the site observed in this crystal structure represents the initial substrate-binding site, with the second binding site located deeper in the transporter barrel (42). This two-site binding model is further supported by the structural analysis of FepA, a close homolog of PfeA, which shows that it binds enterobactin at two locations, one of which is analogous to the PfeA-binding site (50). Fiu and the other TBDTs analyzed lack the extracellular loop structure required to bind their substrates in this external location, and so it is likely that the binding site observed in PfeA and FepA is distinct from other TBDTs. These data suggest that TBDTs share a common substrate-binding site that is analogous to the Fiu external binding site identified by the docking analysis, providing validation of this result.

### The amino acids at the Fiu external binding site are important for iron acquisition in vivo

**Figure 6.**
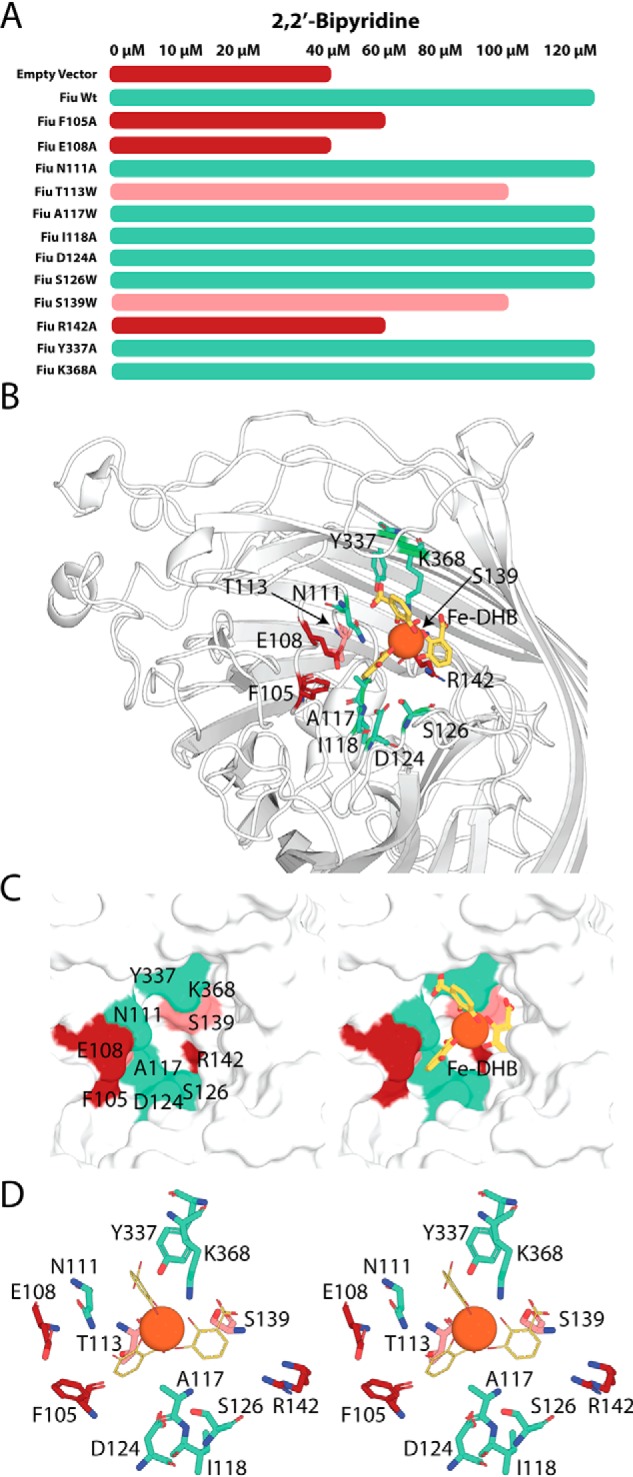
**The effect of mutations in the Fiu external substrate-binding site on the function of Fiu *in vivo*.** *A*, bar graph indicating the effect of Fiu binding site mutations of the ability of pBAD24Fiu to complement *E. coli* BW25113 Δ6. The length of the bar graph indicates the maximum concentration of 2,2′-bipyridine at which growth of the complemented strain was observed on solid LB agar; experimental data are shown in Fig. S5. *B*, stick and cartoon representation of the Fiu extracellular loops, showing the location of the residues in the putative substrate-binding site subjected to mutagenesis. *C*, magnified view of the Fiu substrate-binding site shown in *B*, rendered as a surface model with mutated residues labeled (*left*) and Fe–DHB shown (*right*). *D*, stereo image showing the residues mutated in the Fiu external binding site as a stick representation. The Fe–DHB complex is shown as a *line* and *sphere* representation for DHB and Fe^3+^, respectively. Colors are consistent through all panels and indicate the effect of mutagenesis on Fiu function: *brick red*, inactivation or significant defect in Fiu function; *pink*, minor defection in Fiu function; *green*, no defect in Fiu function.

To determine the role of the amino acids that define the putative Fiu external substrate binding site, we assessed the functionality of variants of Fiu with mutations in this region (Fig. 6 and Fig. S5). Small side chains around the cavity (contributed by alanine, serine, and threonine) were mutated to the bulky amino acid tryptophan to sterically occlude the binding pocket, whereas larger side chains defining the pocket were mutated to alanine. Plasmid-borne constructs of these mutant Fiu proteins were transformed into the *E. coli* BW25113 strain Δ6 (Fig. 1, *B* and *C*). To test for the restoration of iron transport activity, the transformed strains were streaked onto LB agar with 2,2′-bipyridine and scored for growth (Fig. 6 and Fig. S5). Mutation of phenylalanine 105 (F105A), glutamate 108 (E108A), and arginine 142 (R142A) grossly affected the function of Fiu (Fig. 6 and Fig. S5). Fiu(E108A) was nonfunctional, displaying growth identical to the negative control. Fiu(F105A) and Fiu(R142A) displayed minimal complementation (Fig. 6 and Fig. S5). Two other mutations, threonine 113 to tryptophan (T113W) and serine 139 to tryptophan (S139W), also exhibited some defect in function compared with WT Fiu (Fig. 6 and Fig. S5).

It is possible the Fiu mutant variants generated in this experiment may have reduced expression or stability, leading at least partially to the observed phenotypic differences in this assay. However, none of the amino acids mutated play a structural role in Fiu, and the TBDT fold is highly stable, making it relatively unlikely that these mutations would have a major effect on Fiu expression or stability. Based on this assumption, we offer the following analysis. Residues Phe-105 and Glu-108 are located on plug domain loop 1 within the predicted Fe–DHB binding pocket (Fig. 6, *B* and *C*). Phe-105 is ordered in both crystal states and forms a hydrophobic pocket that shelters the aromatic carbons of one of the DHB monomers of the Fe–DHB complex in our docked structure. Glu-108 is disordered in the open state of Fiu and is not within bonding distance of docked Fe–DHB (5.3 Å) but may form interactions with polar substrate groups *in vivo* or generally stabilize the binding pocket (Fig. 6, *B* and *D*). Arg-142 is minimally surface-exposed and unlikely to interact directly with catecholate substrates (Fig. 6, *B* and *C*). However, it is located in plug domain loop 2 and forms hydrogen bonds with the carbonyl groups of amino acids Asp-136 and Gly-138. These interactions stabilize this loop, which defines the lower section of the putative substrate-binding pocket, and this may account for the phenotypic effect of the mutation. Mutation of Asn-111, Tyr-337, and Lys-368, which directly interact with Fe–DHB in our docked structure, did not affect Fiu function in our assay (Fig. 6). This may reflect a lack of precision of our docked model or the fact that single point mutations of substrate-interacting residues may be insufficient to affect substrate import, as reported for other TBDTs (41). Given that several single mutations in this region dramatically effect Fiu function, these data provide evidence that the external binding site identified in our docking is important for Fiu function.

## Discussion

*E. coli* BW25115 possesses three outer-membrane TBDTs known to be responsible for the transport of Fe–catecholate complexes: Fiu, FepA, and Cir (32, 51). In this work, we show that, despite sharing substrates related by a catecholate functional group, Fiu is only distantly related to FepA and Cir. This demonstrates that TBDTs from different lineages have the ability to transport similar substrates, and they may have arrived at this specificity via convergent evolution.

FepA is a high-affinity transporter for enterobactin, a siderophore endogenously produced by *E. coli* (31, 36). Although FepA transports enterobactin, like Fiu and CirA, it is also able to mediate the import of iron in complex with monomeric catecholates, albeit with a lower affinity (32). As Fiu does not transport enterobactin, it is unclear whether its major physiological role is transport of iron in complex with monomeric catecholate molecules or import of a so far unidentified xenosiderophore. In this work, we show that Fiu supports growth of *E. coli* in the absence of an exogenously supplied substrate, likely through import of Fe–catecholate compounds generated through breakdown of enterobactin. However, the ability of Fiu to transport iron under these conditions is inferior to that of FepA and does not support growth under stringent iron limitation. The relatively poor ability of Fiu to transport iron under these conditions suggests that transport of enterobactin breakdown products is a secondary function for this transporter, leaving its high-affinity substrate to be identified. This hypothesis is further supported by our inability to obtain a *bona fide* complex between Fiu and either Fe–DHB or Fe–DHBS, despite identifying conditions that were clearly compatible with substrate crystallization.

The crystal structure and associated analysis we present further reinforce the differences between Fiu and FepA. FepA and its closely related homolog PfeA from *P. aeruginosa* initially bind enterobactin at an external binding site formed by extracellular loops that entirely enclose the entrance to the lumen of the transporter's β-barrel (42, 50). Fiu lacks the extracellular loop structure required for this external binding site, and our docking analysis suggests that it binds its substrate deep in its internal cavity, at a location shared with other TBDTs (38, 41–49).

Structural analysis of Fiu reveals that a number of extracellular loops are selectively ordered and that this selective order is shared among the homologous receptors PiuA and PiuD (30, 33). The observed crystal states may represent conformational transitions the receptor undergoes during substrate binding and transport. Specifically, the external plug loop of Fiu in our structures selectively gates a large cavity in the lumen of the transporter. Our docking shows that this cavity is large enough to accommodate a Fe–catecholate siderophore complex. Additionally, based on predictions of the labile subdomain of the N-terminal plug (37, 39), this cavity is in the path of substrate transport (Fig. 2*C*). This selectively ordered Fiu plug loop may allow its substrate to enter the internal cavity prior to removal of the labile subdomain. If the external plug loop then adopted an ordered state during subdomain removal by TonB, it would prevent channel formation through Fiu during import and prevent nonspecific import of deleterious substances. Despite their significant structural differences and distinct initial binding sites, the essence of this mechanism may be shared with FepA, which possesses a second enclosed binding site deeper in the barrel of the transporter (42, 50).

Gram-negative bacteria can exhibit formidable resistance to antibiotics, largely because of the protective semipermeability of the outer membrane (2, 52, 53). Catecholate-containing antibiotics use molecular mimicry to facilitate their import into bacteria via Fiu and related transporters (25). As these transporters are present in diverse bacterial groups, siderophore-mimicking antibiotics are attractive lead compounds for the development of new therapeutics to treat Gram-negative bacterial infections, representing an area of considerable recent interest for antibiotic development (28, 29, 54). In *A. baumannii* and *P. aeruginosa*, PiuA has been shown to greatly promote susceptibility to the 3,4-dihydroxypyridine–containing sulbactam BAL30072 (30, 55). By investigating the structural basis of substrate binding and import by Fiu, this work will assist with the exploitation of Fiu and related transporters as a conduit for catecholate-containing antibiotics into the bacterial cell.

## Experimental procedures

### TBDT phylogeny construction, Fiu homolog search, and clustering analysis

To determine the phylogenetic relationship between Fiu and TBDTs of know structure and/or function, a subset of TBDT sequences were selected, and these sequences were obtained from the NCBI database. Sequences were aligned using the Clustal algorithm, and the alignment was utilized to build a bootstrapped phylogenetic tree (100 repetitions), which was visualized using the FigTree software (56).

A HMMER search using the Fiu sequence from *E. coli* BW25113 as the search query was established using the stable and unbiased proteome dataset RP55 (57, 58). The search was restricted to sequences with an E-value of less than 1e−75, limiting the outcome to 502 sequences (Table S1). For sequence clustering, classification used all-against-all BLAST clustering based on pairwise similarities and visualized with CLANS (59), with an E-value cutoff of 1 × 10^−120^. To assess the similarity of sequences identified in this search to other TBDTs present in *E. coli* BW25113, sequences for FhuA, FecA, CirA, FepA, BtuB, YddB, and YncD were added to this dataset, and clustering was performed with an E-value cutoff of 1 × 10^−120^.

### Construction of a multiple TBDT knockout E. coli BW25113 strain

*E. coli* BW25113 mutants were created using the λ-red system (60). Kanamycin resistance cassettes flanked by 300 bp of genomic DNA either side of the genes encoding TBDTs of interest were amplified using specific mutants from the *E. coli* mutant Keio collection (61) as templates. Primers utilized are summarized in Table S7.

The host strain *E. coli* BW25113 was transformed with the λ-red recombinase plasmid pKD46 (60), grown at 30 °C (LB broth and 100 μg·ml^−1^ ampicillin) to an *A*_600 nm_ of 0.1 before λ recombinase was induced by addition of 0.2% l-arabinose. Thereafter, cultures were grown at 30 °C until *A*_600 nm_ 0.6–0.8 and transformed using the room temperature electroporation method (62). Briefly, bacterial cells were isolated by centrifugation at 3000 × *g* for 3 min and washed twice with a volume of sterile 10% glycerol equal to the volume of the culture used. Cells were then resuspended in 10% glycerol to a volume of 1:15 that of the original culture. 100–500 ng of PCR-amplified Kan^R^ KO cassette for the gene of interest was then added to 100 μl of the resuspended bacteria, and the mixture was electroporated. 1 ml of LB broth was added to the cells after electroporation, and the culture was recovered at 37 °C for 1 h before plating onto LB agar and 30 μg·ml^−1^ kanamycin. PCR was used to validate that colonies did indeed have the Kan^R^ cassette in place of the gene of interest.

To remove the Kan^R^ cassette, deletion mutant strains were transformed with the plasmid pCP20 (63) containing the “flippase cassette.” Cells were grown under either ampicillin (100 μg·ml^−1^) or chloramphenicol (30 μg·ml^−1^) selection to maintain the plasmid. For removal of the Kan^R^ cassette, a single colony of the mutant strain was used to inoculate 1 ml of LB broth (no selection). The culture was grown overnight at 43 °C to activate expression of the flippase gene. This culture was then subject to 10-fold serial dilution in sterile LB and plated onto LB agar with no selection. The resulting colonies were patched onto LB agar containing kanamycin, chloramphenicol, or no selection. PCR was used to validate colonies unable to grow on kanamycin or chloramphenicol; those able to grow in the absence of selection did indeed represent successful removal of the Kan^R^ cassette. This process was repeated sequentially to derive strains multiply defective in up to six TBDT receptors. The order of deletion was ΔFhuA, ΔFecA, ΔCirA, ΔFepA, ΔFhuE, and then ΔFiu. Mutant strains created in the process were designated TBDT Δ1, Δ2, Δ3, Δ4, Δ5, and Δ6, based on the number of receptors deleted. Strains created are listed in Table S8.

### Testing the growth of E. coli BW25113 TBDT deletion strains under iron-limiting conditions

*E. coli* BW25113 deletion strains (Δ4, Δ5, and Δ6) grew poorly on LB agar. To ameliorate this phenotype, all mutant strains were maintained on LB agar and 250 μm Fe(II)SO_4_. All deletion strains grew well under these conditions. To test the ability of mutant strains to grow under iron-limiting conditions, strains were grown in LB broth until stationary phase. Cells were harvested from 0.5 ml of this stationary-phase culture, and the supernatant was removed. Cells were resuspended in 0.5 ml of 1× M9 salts, and a minimal quantity of this suspension was streaked onto LB agar containing 0–150 μm 2′2-bipyridine. Plates were incubated at 37 °C overnight, and growth was observed and scored.

### Complementation of TBDT receptor mutants with WT and mutant Fiu

The ORF for Fiu, including the sequence encoding the signal peptide, was amplified from *E. coli* BW25113 by PCR (Table S7) and cloned into the pBAD24 plasmid at EcoRI and HindIII restriction sites. The resulting vector, designated pBAD24Fiu, was then transformed into *E. coli* BW25113 Δ6 and maintained using 100 μg·ml^−1^ ampicillin. To test for complementation, *E. coli* BW25113 TBDTΔ6 pBAD24Fiu was streaked onto LB agar and 0.2% arabinose, 100 μg·ml^−1^ ampicillin, and 0–120 μm 2,2′-bipyridine. Growth under these conditions was compared with that of *E. coli* BW25113 Δ6 containing pBAD24 as a vector control. Mutations of the putative Fiu substrate-binding site were created via whole-plasmid mutagenesis using pBADFiuCom as the starting vector (64). The mutations were introduced using the primer sequences provided in Table S7. The sequence of the pBAD24Fiu template and introduction of the specified mutations in the resultant plasmids were confirmed by Sanger sequencing. Mutant plasmids were transformed into *E. coli* BW25113 TBDTΔ6, maintained, and tested for function as described above for pBAD24Fiu.

### Protein expression and purification

DNA encoding the mature form of Fiu lacking the signal peptide was amplified from *E. coli* BW25113 using the primers shown in Table S7. NcoI and XhoI restriction sites incorporated into the primers were used to clone the DNA fragment into a modified pET20b vector with a 10× N-terminal His tag followed by a TEV cleavage site. The resulting plasmid was transformed into *E. coli* BL21 (DE3) C41 cells, and protein expression was induced in cultures grown in terrific broth (12 g of tryptone, 24 g of yeast extract, 61.3 g of K_2_HPO_4_, 11.55 g of KH_2_PO_4_, and 10 g of glycerol) with 100 mg·ml^−1^ ampicillin for selection. Cultures were grown at 37 °C until *A*_600_ of 1.0, induced with 0.3 mm isopropyl 1-thio-β-d-galactopyranoside, and grown for a further 14 h at 25 °C. Cells were harvested by centrifugation, lysed with a cell disruptor (Emulseflex) in lysis buffer (50 mm Tris, 200 mm NaCl, and 2 mm MgCl_2_) plus 0.1 mg·ml^−1^ lysozyme, 0.05 mg·ml^−1^ DNase1, and Complete protease mixture inhibitor tablets (Roche). The resulting lysate was clarified by centrifugation at 20,000 × *g* for 10 min, the supernatant was then centrifuged for a further 1 h at 160,000 × *g* to isolate membranes. The resultant supernatant was decanted, and the membrane pellet was suspended in lysis buffer using a tight-fitting homogenizer. When homogenized, the membrane fraction was solubilized by addition of 10% Elugent (Santa Cruz Biotechnology) and incubated with gentle stirring at room temperature for 20 min. Solubilized membrane proteins were clarified by centrifugation at 20,000 × *g* for 10 min. The supernatant was applied to Ni-agarose resin equilibrated in Ni binding buffer (50 mm Tris, 500 mm NaCl, 20 mm imidazole, and 0.03% dodecyl maltoside (DDM) (pH 7.9)). The resin was washed with 10–20 column volumes of Ni binding buffer before elution of the protein with a step gradient of 10%, 25%, 50%, and 100% Ni gradient buffer (50 mm Tris, 500 mm NaCl, 1 m imidazole, and 0.03% DDM pH 7.9)). Fiu eluted at 50% and 100% gradient steps. Eluted fractions containing Fiu were pooled and applied to a 26/600 S200 Superdex size exclusion column equilibrated in SEC buffer (50 mm Tris, 200 mm NaCl, and 0.03% DDM pH 7.9)). To exchange Fiu into octyl β-d-glucopyranoside (βOG) for crystallographic analysis, fractions from SEC containing Fiu were pooled and applied to Ni-agarose resin equilibrated in βOG buffer (50 mm Tris, 200 mm NaCl, and 0.8% octyl β-d-glucopyranoside (pH 7.9)). The resin was washed with 10 column volumes of βOG buffer before elution with βOG buffer and 250 mm imidazole. Fractions containing Fiu were pooled, and 1 mg·ml^−1^ His_6_-tagged TEV protease and 1 mm DTT were added. This solution was then dialyzed against βOG buffer at 4–6 h at 20 °C to allow TEV cleavage of the His_10_ tag and removal of excess imidazole. The solution was then applied to Ni-agarose resin to remove TEV protease and the cleaved polyhistidine peptide. The flow-through containing Fiu from this step was collected concentrated to 14 mg·ml^−1^ in a 30-kDa cutoff centrifugal concentrator, snap-frozen, and stored at −80 °C.

### Protein crystallization, data collection, and structure solution

Purified Fiu in βOG buffer was screened using commercially available crystallization screens (∼600 conditions). Hexagonal crystals formed in the JCSG screen in 1 m LiCl, 20% PEG 6000, and 0.1 m trisodium citrate (pH 4.0) (65). These crystals were looped and the drop solution was removed, and they were flash-cooled and stored in liquid N_2_. These crystals diffracted poorly (>3.3 Å) and suffered from considerable anisotropy. To improve diffraction, Fiu under the above condition was subjected to an additive screen (Hampton Research). Hexagonal crystals grew with many additives; however, in the presence of 5% polypropylene glycol P400 (PPG 400), flat, diamond-shaped plates formed. These crystals were looped, the mother liquor was removed by wicking, and they were flash-cooled and stored in liquid N_2_ at 100 K. Data were collected at the Australian Synchrotron, with crystals diffracting to 2.1 Å in the space group C222_1_. Despite relatively low sequence identity (33%) between Fiu and the catecholate receptor PiuA from *A. baumannii* (PDB code 5FP1), a molecular replacement solution was obtained using Phaser, with the crystal structure of the PiuA receptor as a starting model (30, 66). The model was built and refined using the Phenix package and Coot (67, 68). The majority of the Fiu polypeptide chain could be modeled into the available density; however, loops 7–9 and the plug domain loop were disordered in this structure. The Fiu model from these crystals was designated crystal state 1.

To obtain Fiu in additional crystal states, purified Fiu in βOG buffer with 5% PPG 400 was rescreened for crystallization (∼600 conditions). Fiu crystallized under multiple addition conditions in the presence of PPG 400. Using crystals looped and frozen directly from these screens, the structure of Fiu was solved in two further crystal forms: the *P1* form at 2.9 Å in 20% PEG 3350, 0.2 m Na_2_ malonate, and 0.1 m BisTris propane (pH 8.5) and the C2_1_ form at 2.5 Å in 0.1 m Tris, 20% PEG 6000, and 0.2 m NaCl (pH 8.0). In these crystal forms, all loops of Fiu were ordered and conformationally analogous, allowing complete tracking of the Fiu sequence amino acids 50–760. Fiu modeled from these crystals was designated crystal state 2. Cocrystallization between Fiu and Fe–DHB or Fe–DHBS was performed by adding 300 μm to 1 mm of these compounds to purified Fiu in βOG buffer with or without 5% PPG 400 to at a final concentration of ∼100 μm (8 mg/ml). Crystal screening was performed as above. Crystals were harvested directly from screening trays, mother liquor was removed by wicking, and crystals were cryocooled in liquid N_2_ at 100 K. For soaking experiments, Fiu crystals from crystal states 1 and 2 were transferred to crystallization solution containing 1 mm Fe–DHB or Fe–DHBS and incubated in this solution for 1–5 min prior to cryocooling. Crystallization of Fiu, 5% PPG 400, and 1 mm Fe–DHB yielded purple crystals in the JCSG screen (65) condition (0.1 m Tris (pH 8.5), 20% (w/v) PEG 8000, and 0.2 m MgCl_2_). These crystals were looped, wicked to remove mother liquor, and flash frozen as above. These crystals diffracted modestly, with the best crystal diffracting anisotropically to 3.2–5.9 Å. Molecular replacement with refined Fiu was performed on this dataset to identify the location of the Fe–DHB complex. RMSD calculations were performed using the RMSD tool in the Align command in PyMOL.

### In silico docking of Fe–DHB with the Fiu crystal structure

To determine potential ligand bindings sites in the Fiu crystal structure, an *in silico* docking approach was applied using Autodock Vina in the Chimera software package (40, 69). Coordinates for iron in complex with three molecules of 2,3-dihydroxybenzoic acid (Fe–DHB) was obtained from PDB code 3U0D, the structure of human Siderocalin bound to the bacterial siderophore 2,3-DHBA. Coordinates for Fiu were taken from molecule A of the C2_1_ crystal form in crystal state 2. Ligand and Fiu coordinates were imported into Chimera and optimized using the Dock Prep utility. Docking was performed using the Autodock Vina dialog, and two box sizes were utilized for docking (box 1 = 46.5, 56.0, 45.5 Å; box 2 = 46.5, 35.2, 45.5 Å). Box 1 encompassed the entire extracellular portion of Fiu and all cavities accessible from the extracellular environment. Box 2 excluded the large cavity in Fiu, gated by the extracellular plug domain. A total of nine binding modes were sought for each docking run, with search exhaustiveness of between eight and 300 and a maximum energy difference of 3 kcal/mol. Docking solutions did not differ significantly as a result of changes to search exhaustiveness. Docking solutions were inspected visually, and the highest-rated solution was used for the main figures and for “Discussion.”

### Analysis of TBDT ligand binding sites

The PDB was searched manually for structural coordinates of TBDT in complex with substrate compounds. TBDT receptor complexes were aligned to the crystal structure of Fiu (Table S6) based on the TBDT chain using the Super command in PyMOL. The location bound substrates were determined by manual inspection in PyMOL. The location and volume of Fiu substrate binding cavities were estimated using CASTp (70).

## Author contributions

R. G. and T. L. conceptualization; R. G. and T. L. resources; R. G. data curation; R. G. formal analysis; R. G. and T. L. funding acquisition; R. G. investigation; R. G. visualization; R. G. methodology; R. G. writing-original draft; R. G. project administration; R. G. and T. L. writing-review and editing; T. L. supervision.

## Supplementary Material

Supporting Information

## Footnotes

3 The abbreviations used are:

TBDT: TonB-dependent transporter
Fiu: ferric iron uptake
DHBS: 2,3-dihydroxybenzoyl-l-serine
RMSD: root mean square deviation
DHB: 2,3-dihydroxybenzoic acid
TEV: tobacco etch virus
DDM: dodecyl maltoside
βOG: octyl β-d-glucopyranoside
PPG: polypropylene glycol
LB: lysogeny broth.
