# Supplementary material for: The structure of the bacterial iron–catecholate transporter Fiu suggests that it imports substrates via a two-step mechanism
Source: J Biol Chem. 2019 Nov 11;294(51):19523–34. doi: 10.1074/jbc.RA119.011018 (PMC6926462; doi:10.1074/jbc.RA119.011018)
Supplement: Supporting Information [file supp_294_51_19523__index.html]

The structure of the bacterial iron–catecholate transporter Fiu suggests that it imports substrates via a two-step mechanism — The structure of Fiu suggests 2-step import — The structure of the bacterial iron–catecholate transporter Fiu suggests that it imports substrates via a two-step mechanism — The structure of Fiu — Supporting Information 

# The structure of the bacterial iron–catecholate transporter Fiu suggests that it imports substrates via a two-step mechanism

## Supporting Information

- Supporting Information (to be published online) - Table S1
- Supporting Information (to be published online) - Atomic Coordinates and Structural Data
- Supporting Information (to be published online) - Supporting Information - Figures and Tables
