# Supplementary material for: The structure of the bacterial iron–catecholate transporter Fiu suggests that it imports substrates via a two-step mechanism
Source: J Biol Chem. 2019 Nov 11;294(51):19523–34. doi: 10.1074/jbc.RA119.011018 (PMC6926462; doi:10.1074/jbc.RA119.011018)
Supplement: Supporting Information [file supp_RA119.011018_155766_2_supp_426544_q0t1dk.pdf]

SUPPORTING INFORMATION

Digital Files:

Table S1 a list of sequences homologous to Fiu identified in the HMMER search (Table S1.xlsx)

Structural Coordinates for Fiu docked with Fe-DHB (DHB_Dock_Run1.pdb, DHB_DockRun2.pdb, Fiu_Dock_Receptor.pdb)

Atomic coordinates and experimental data for Fiu Fe-DHB cocrystal (Fiu_DHB.pdb, Fiu_DHB.mtz, Fiu_DHB_refine.mtz)

Supporting Figures:

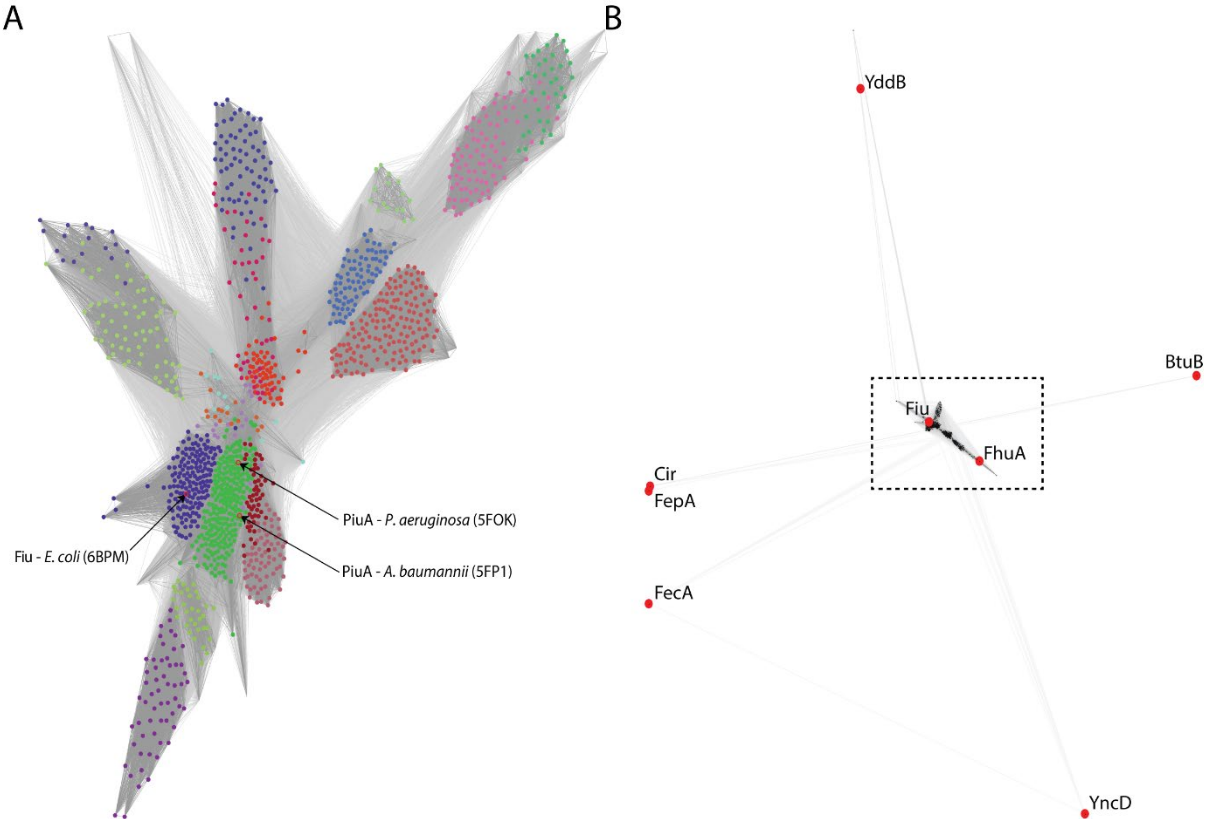

Figure S1 CLANS clustering of Fiu homologs identified by HMMER search and other TBDTs from *E. coli*.

(A) CLANS clustering analysis (59) of Fiu homolog sequences identified by HMMER search (Table S1), location of the points corresponding to Fiu and PiuA are indicated. Sequences in (B) are spiked with TBDT sequences from *E. coli* BW25113 to show the relationship between these receptors and the Fiu homologs identified in the search.

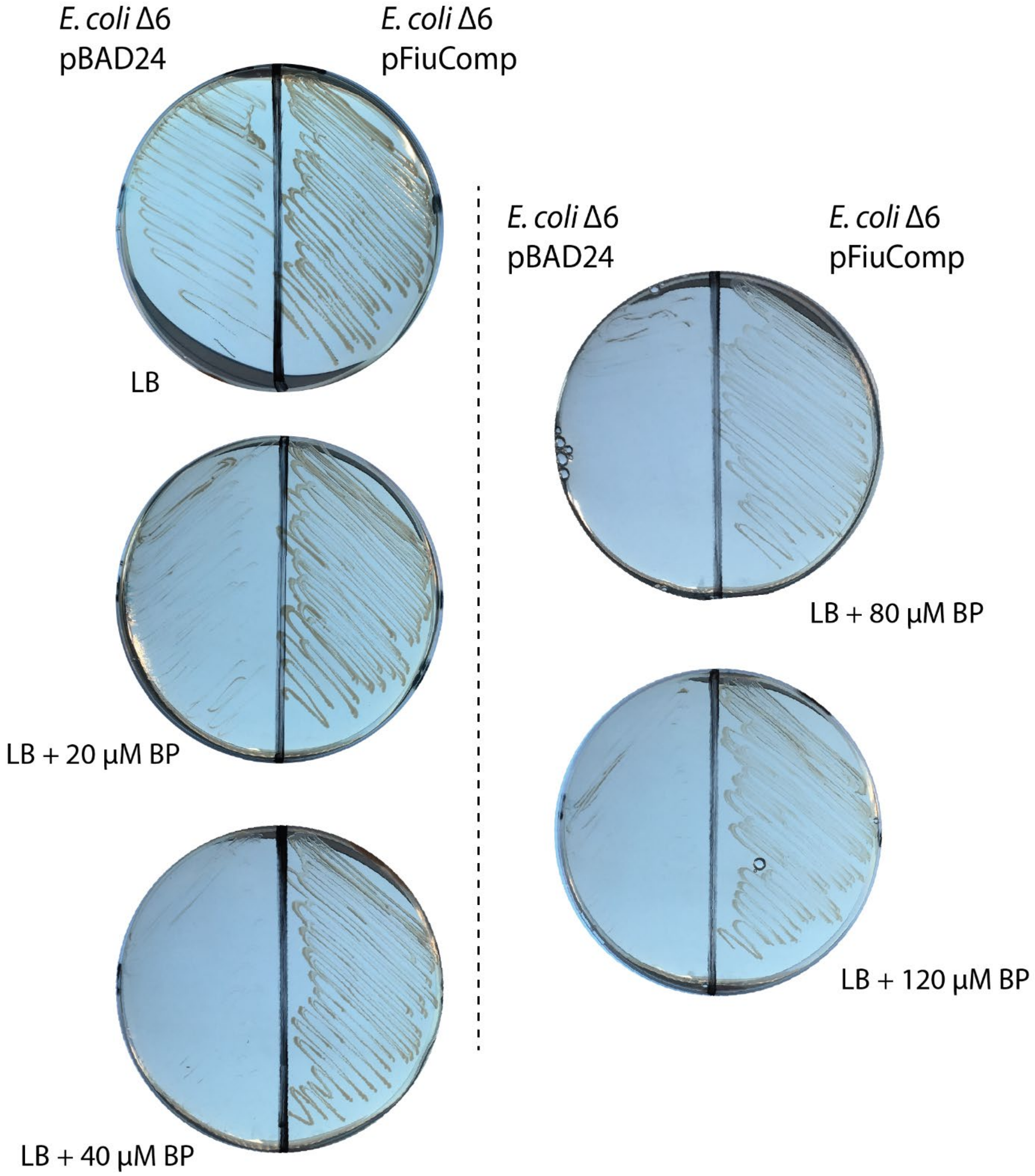

Figure S2 Complementation of *E. coli* BW25113 TBDTΔ6 with plasmid derived Fiu

Growth of *E. coli* BW25113 TBDTΔ6 with complementation vector (pBAD24Fiu) or vector control (pBAD24) on LB agar with 0-120 µM 2,2'-Bipryidine (BP). Vector control is unable to grow at a concentration of BP > 20 µM, while the complemented strain can grow at a concentration up to BP = 120 µM.

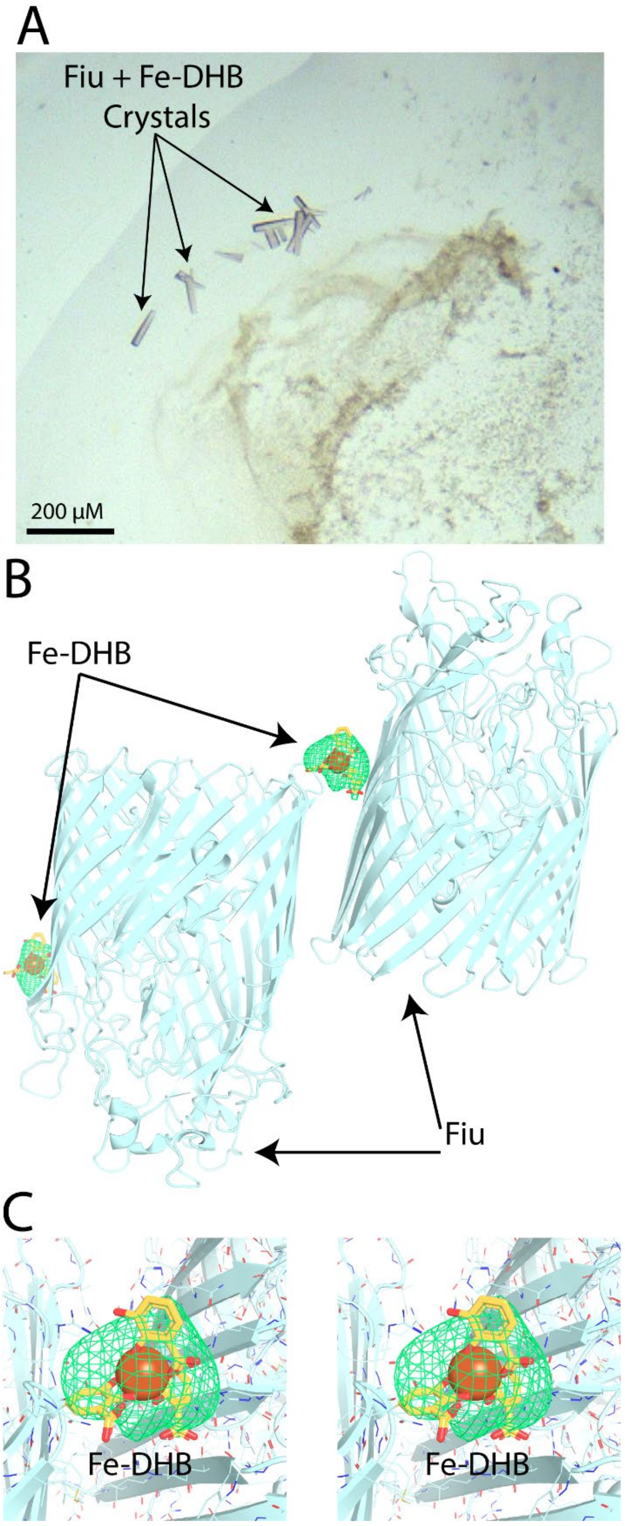

Figure S3 Co-crystallisation between Fiu and Fe-DHB complex
(A) Crystals of Fiu obtained in the presence of Fe-DHB, with a violet color characteristic of a Fe-catecholate complex. (B) The resultant asymmetric unit from these poorly diffracting crystals (anisotropic 3.2-5.9 Å) contained 2 molecules of Fiu in complex with 2 Fe-DHB complexes. Fiu is shown in cartoon representation, while Fe-DHB is shown as a stick/sphere model modeled into Fo-Fc density contoured to 3 σ. (C) A zoomed stereo view of the central Fe-DHB complex from panel B, with Fiu sidechains shown as lines. Fe-DHB is coordinated by a patch of positively charged residues on one Fiu molecule.

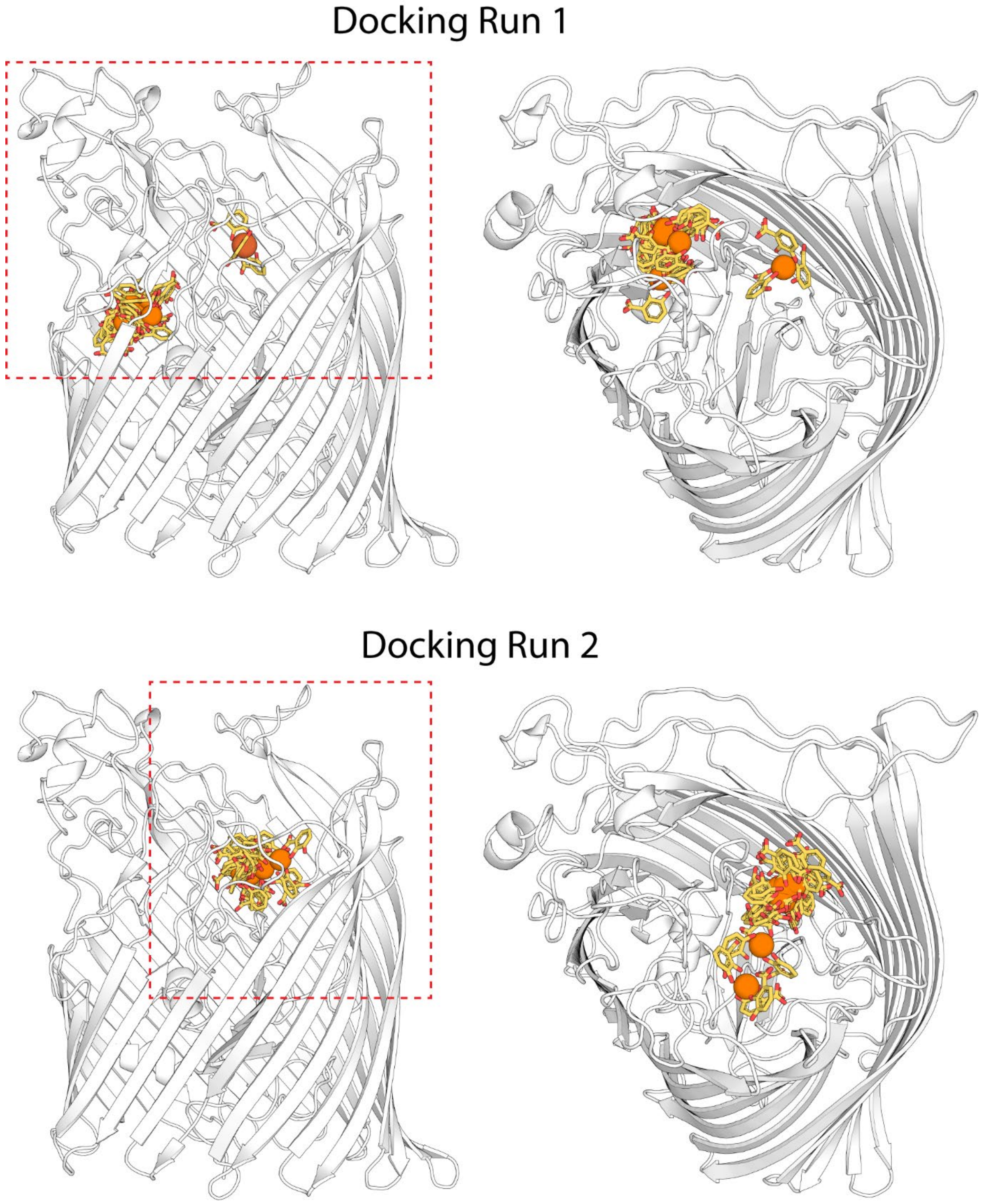

Figure S4 All docking modes obtain between Fiu and Fe-DHB

All 9 docking modes obtained for docking runs 1 and 2, shown with Fe-DHB as a stick/sphere model with Fiu with a cartoon representation. The dashed red box shows the docking search area along the Z-axis relative to the membrane. In both docking runs the majority of binding modes, including the top-ranked mode, cluster at a single location.

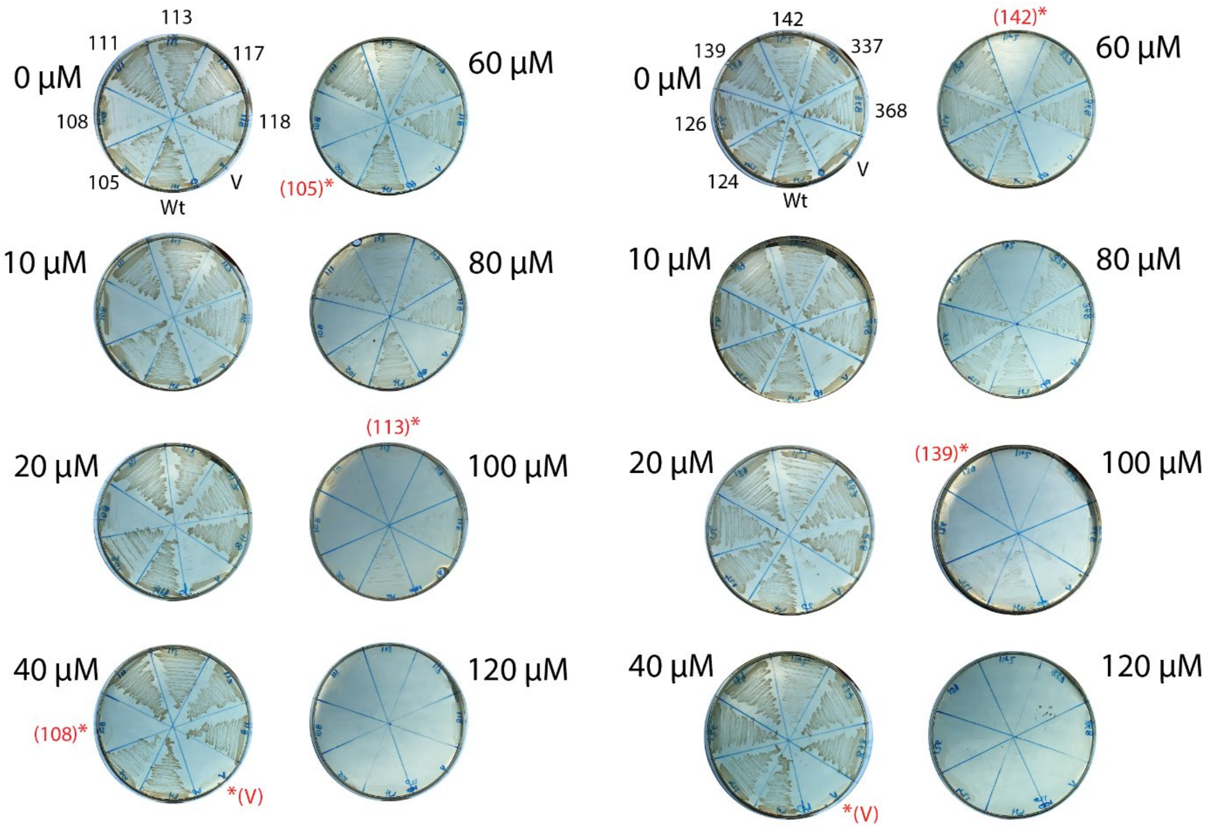

Figure S5 The effect of substrate binding site mutations on *in vivo* function of Fiu

The growth of *E. coli* BW25113 containing the complementation vector (Wt), vector control (V) or complementation vector mutants (numbers) on LB agar with 0-120 μM 2,2'-bipyridine (BP). Mutant numbers represent the position of the amino acids mutated (F105A, E108A, N111A, T113W, A117W, Y118A, D124A, S126W, S139W, R142A, Y337, L368A). Starred sections show the maximum BP concentration at which the corresponding mutated construct or control is able to grow.

Supporting Tables:

Table S2 Sequence identity matrix of a selection of Fiu homologs from bacteria belonging to Enterobacteriaceae.

	P. dispersa	*P. wallisii*	*P. sp. (strain At-9b)*	*M. sp. MFB070*	*K. pneumoniae DSM 30104*	*P. gergoviae*	*E. coli (strain K12)*	*E. coli ISC11*	*L. adecarboxylata*	*R. ornithinolytica*	*K. ascorbata ATCC 33433*	*S. blattae DSM 4481*	*S. sp. M24T3*	*S.sp. Leaf50*	*E. gerundensis*
Pantoea dispersa	100	94	91	86	85	80	79	79	80	80	76	74	78	79	81
Pantoea wallisii	94	100	88	84	84	79	78	78	78	78	74	73	77	78	80
Pantoea sp. (strain At-9b)	91	88	100	86	84	79	79	80	80	81	78	74	78	80	78
Mangrovibacter sp. MFB070	86	84	86	100	83	80	77	79	80	79	76	72	78	79	78
Klebsiella pneumoniae DSM 30104	85	84	84	83	100	79	77	76	78	77	75	72	75	77	77
Pluralibacter gergoviae	80	79	79	80	79	100	74	73	74	73	71	69	72	73	74
Escherichia coli (strain K12)	79	78	79	77	77	74	100	89	86	83	82	77	77	77	78
Escherichia coli ISC11	79	78	80	79	76	73	89	100	87	86	84	76	80	78	79
Leclercia adecarboxylata	80	78	80	80	78	74	86	87	100	85	82	75	77	78	79
Raoultella ornithinolytica	80	78	81	79	77	73	83	86	85	100	83	77	78	79	80
Kluyvera ascorbata ATCC 33433	76	74	78	76	75	71	82	84	82	83	100	74	78	78	75
Shimwellia blattae DSM 4481	74	73	74	72	72	69	77	76	75	77	74	100	73	72	73
Serratia sp. M24T3	78	77	78	78	75	72	77	80	77	78	78	73	100	86	77
Serratia sp. Leaf50	79	78	80	79	77	73	77	78	78	79	78	72	86	100	78
Erwinia gerundensis	81	80	78	78	77	74	78	79	79	80	75	73	77	78	100

Table S3 Data collection and refinement statistics for fully refined Fiu crystal structures

	Crystal State 1	Crystal State 2	
**Data Collection**[a]			
Space Group	*C222(1)*	*C2(1)*	*P1*
Cell Dimensions			
a, b, c (Å)	107.93, 148.94, 105.06	191.43, 75.67, 136.87	81.67, 133.16, 135.10
α, β, γ (°)	90, 90, 90	90, 91.89, 90	101.5, 107.42, 92.88
Wavelength	0.976	0.976	0.976
Resolution (Å)	48.00-2.10 (2.16-2.10)	49.34-2.50 (2.56-2.50)	50.31-2.9 (2.95-2.90)
R_{merge}	0.152 (0.886)	0.156 (1.026)	0.222 (1.120)
R_{pim}	0.058 (0.349)	0.111 (0.740)	0.151 (0.802)
I/s(*I*)	10.5 (2.4)	5.4 (1.1)	3.6 (1.1)
$CC_{1/2}$	0.997 (0.811)	0.988 (0.536)	0.963 (0.491)
Completeness (%)	100.0 (100.0)	100.0 (100.0)	99.0 (98.5)
Redundancy	7.7 (7.4)	3.6 (3.5)	3.7 (3.7)
Reflections	49686 (4026)	68066 (4595)	116009 (5714)
**Refinement statistics**			
R_{work}/R_{free}	17.4/22.0	19.9/25.9	21.8/26.9
No. atoms			
Protein	5025	10823	21644
Ligand / ions	146	235	40
R.m.s deviations			
Bond lengths (Å)	0.007	0.007	0.008
Bond angles (°)	0.817	0.999	1.09
PDB ID	6BPN	6BPM	6BPO

[a] Values in parentheses are for highest-resolution shell.

Data from one crystal was collected for each structure

Table S4 Data collection and refinement statistics for partially refined low resolution Fiu/Fe-DHB cocrystal

	**Fe-DHB Co-crystal**
**Data Collection[a]**	
Space Group	*P2(1)2(1)2(1)*
Cell Dimensions	
a, b, c (Å)	72.60, 129.32, 200.77
α, β, γ (°)	90, 90, 90
Wavelength	0.976
Resolution (Å)	49.21-3.23 (3.43-3.23)
**Anisotropy correction[b]**	
Resolution truncation (reflections with F/σ < 3.0 discarded)	
a, b*, c* (Å)*	5.58, 5.88, 3.23
Reflections discarded	
Original, discarded, final	62488, 51875, 10613
Completeness after truncation (%)	
49.21-5.71 Å, 5.71-4.54 Å, 4.54-3.23 Å	99.8, 50.0, 10.0
**Refinement statistics**	
R_{work}/R_{free}	31.1/34.9
No. atoms	
Protein	10854
Ligand / ions	0
R.m.s deviations	
Bond lengths (Å)	0.012
Bond angles (°)	1.377

[a] Values in parentheses are for the highest-resolution shell.

[b] Correction applied using 'Diffraction Anisotropy Server'[1]

Table S5 Fiu Fe-DHB Autodock docking statistics

Mode	Affinity (kcal/mol)	Distance from RMSD l.b.	Best Mode RMSD u.b.
		Docking run 1	
1	-10.6	0.00	0.00
2	-10.0	5.08	8.56
**3**	**-9.8**	**15.87**	**18.46**
4	-9.7	2.51	7.08
5	-9.7	2.49	2.90
6	-9.7	1.75	5.92
7	-9.6	2.78	6.48
8	-9.6	2.45	7.08
9	-9.6	1.35	6.91
		Docking run 2	
**1**	**-9.8**	**0.00**	**0.00**
2	-9.0	5.96	11.28
3	-9.0	1.39	6.35
4	-9.0	11.14	15.41
5	-8.9	1.99	2.54
6	-8.9	2.66	6.46
7	-8.9	2.33	6.83
8	-8.8	2.00	2.41
9	-8.8	1.95	6.03

Table S6 The crystal structures of TBDT-substrate complexes utilized for binding site analysis.

PDB Code	Organism	Receptor	Substrate
1BY5	*E. coli*	FhuA	Ferrichrome
1KMP	*E. coli*	FecA	Ferric Citrate
1NQH	*E. coli*	BtuB	Cyanocobalamin (Vitamin B12)
3QLB	*Pseudomonas fluorescens*	FptA	Ferri-enantiopyochelin
2IAH	*Pseudomonas aeruginosa*	FpvA	Ferri-pyoverdine
4RDT	*Neisseria meningitidis*	ZnuD	Zinc ion
6I96	*P. aeruginosa*	FoxA	Ferroxiamine
6E4V	*E. coli*	FhuE	Coprogen
6I2J	*P. aeruginosa*	PfeA	Enterobactin
6FOK	*P. aeruginosa*	OprC	Copper Ion
6H7F	*Acinetobacter baumannii*	BauA	Acintobactin
4AIQ	*N.meningitidis*	FrpB	Ferric Ion

Table S7 Oligonucleotide primers used in this study

Primer Name	Sequence (5'-3')	Purpose
Fiu Expr F	CCATCCCATGGGTGCCGAAGGGCAAACTAACGC	Fiu amplication for pET20b protein expression
Fiu Expr R	CCATCCTCGAGTCAGAAATGCATATTGGCTGTGAG	Fiu amplication for pET20b protein expression
ΔFepA F	AGCGTGTTGCAGTTGTGCGTA	FepA KO cassette amplification
ΔFepA R	CCGGGCCATGTTTCGACTG	FepA KO cassette amplification
ΔFhuA F	CAACAGCAACCTGCTCAGCAA	FhuA KO cassette amplification
ΔFhuA R	CAAACGCTTTGCTGCTCCAGC	FhuA KO cassette amplification
ΔFhuE F	CAGCGAAATGGCACCGGCT	FhuE KO cassette amplification
ΔFhuE R	CGCCACACTGCGAGTATAGA	FhuE KO cassette amplification
ΔCirA F	AGGACAAAAATGCATGGCTGGAAT	CirA KO cassette amplification
ΔCirA R	GAAATAAGTTTCCTCCCTTCCTTG	CirA KO cassette amplification
ΔFecA F	TTCCGACGGACTGCCACGG	FecA KO cassette amplification
ΔFecA R	ACCGCTGGGTGAGGTGATAG	FecA KO cassette amplification
Δfiu F	ACAGCGGCGTGGAAAGGGTA	Fiu KO cassette amplification
Δfiu R	GTTCGGTACTGCAACCATCTAC	Fiu KO cassette amplification
Fiu Comp F	GTACGGAATTCATGGAAAACAATCGCAATTTCCCTG	Fiu amplification for pBAD24 complementation
Fiu Comp R	GTACGAAGCTTTCAGAAATGCATATTGGCTGTGAGC	Fiu amplification for pBAD24 complementation
Fiu F105A F	GGCGTGGGTGCGTTTGCTGCGGGTGAGAACGGT	Fiu Mutagenesis F105A
Fiu F105A R	ACCGTTCTCACCCGCAGCAAACGCACCCACGCC	Fiu Mutagenesis F105A
Fiu E108A F	GCGTTTTTTGCGGGTGCGAACGGTAACTCCACCA	Fiu Mutagenesis E108A
Fiu E108A R	TGGTGGAGTTACCGTTCGCACCCGCAAAAAACGC	Fiu Mutagenesis E108A
Fiu N111A F	CGGGTGAGAACGGTGCCTCCACCACTGGCGAC	Fiu Mutagenesis N111A
Fiu N111A R	GTCGCCAGTGGTGGAGGCACCGTTCTCACCCG	Fiu Mutagenesis N111A

Fiu T113W F	CGGGTGAGAACGGTAACTCCTGGACTGGCGACGCCATTTATATG	Fiu Mutagenesis T113W
Fiu T113W R	CATATAAATGGCGTCGCCAGTCCAGGAGTTACCGTTCTCACCCG	Fiu Mutagenesis T113W
Fiu A117W F	GGTAACTCCACCACTGGCGACTGGATTTATATGCGTGGTGCC	Fiu Mutagenesis A117W
Fiu A117W R	GGCACCACGCATATAAATCCAGTCGCCAGTGGTGGAGTTACC	Fiu Mutagenesis A117W
Fiu I118A F	CACCACTGGCGACGCCGCTTATATGCGTGGTGCC	Fiu Mutagenesis Y118A
Fiu I118A R	GGCACCACGCATATAAGCGGCGTCGCCAGTGGTG	Fiu Mutagenesis Y118A
Fiu D124A F	GCCATTTATATGCGTGGTGCCGCTACCTCTAACAGTATTTATA,	Fiu Mutagenesis D124A
Fiu D124A R	TATAAATACTGTTAGAGGTAGCGGCACCACGCATATAAATGGC	Fiu Mutagenesis D124A
Fiu S126W F	ATGCGTGGTGCCGATACCTGGAACAGTATTTATATTGATGGCA	Fiu Mutagenesis S126W
Fiu S126W R	TGCCATCAATATAAATACTGTTCCAGGTATCGGCACCACGCAT	Fiu Mutagenesis S126W
Fiu S139W F	CATTCGCGATATCGGCTGGGTCTCGCGCGACACCT	Fiu Mutagenesis S139W
Fiu S139W R	AGGTGTCGCGCGAGACCCAGCCGATATCGCGAATG	Fiu Mutagenesis S139W
Fiu R142A F	TATCGGCAGCGTCTCGGCCGACACCTTCAATACCGA	Fiu Mutagenesis R142A
Fiu R142A R	TCGGTATTGAAGGTGTCGGCCGAGACGCTGCCGATA	Fiu Mutagenesis R142A
Fiu Y337A F	CGCGCGTAAAGCAGGATGCCCTGATGACGGCGATTATG	Fiu Mutagenesis Y337A
Fiu Y337A R	CATAATCGCCGTCATCAGGGCATCCTGCTTTACGCGCG	Fiu Mutagenesis Y337A
Fiu L368A F	TGGTCACGCACGGCGAATACCGCAGATGTGAGTAATAAAATTC	Fiu Mutagenesis L368A
Fiu L368A R	GAATTTTATTACTCACATCTGCGGTATTCGCCGTGCGTGACCA	Fiu Mutagenesis L368A

Table S8 Strains and plasmids used or generated in this study

Strain or plasmid	Relevant genotype and description	Reference or source
Escherichia coli BW25113 Wt	F-, DE(araD-araB)567, lacZ4787(del)::rrnB-3, LAM-, rph-1, DE(rhaD-rhaB)568, hsdR514	[2]
Escherichia coli BW25113 TBDT Δ1	As Wt + FhuA(del)	This study
Escherichia coli BW25113 TBDT Δ2	As Wt + FhuA(del),FecA(del)	This study
Escherichia coli BW25113 TBDT Δ3	As Wt + FhuA(del),FecA(del),CirA(del)	This study
Escherichia coli BW25113 TBDT Δ4	As Wt + FhuA(del),FecA(del),CirA(del),FepA(del)	This study
Escherichia coli BW25113 TBDT Δ5	As Wt + FhuA(del),FecA(del),CirA(del),FepA(del),FhuE(del)	This study
Escherichia coli BW25113 TBDT Δ6	As Wt + FhuA(del),FecA(del),CirA(del),FepA(del),FhuE(del),Fiu(del)	This study
p20bFiu	pET20b + Fiu -minus signal peptide ligated at NcoI/XhoI sites	This study
pBAD24Fiu	pBAD24 + Fiu ligated at EcoRI/HindIII sites	This study
pBAD24FiuF105A	As pBAD24Fiu with mutation as labelled	This study
pBAD24FiuE108A	As pBAD24Fiu with mutation as labelled	This study
pBAD24FiuN111A	As pBAD24Fiu with mutation as labelled	This study
pBAD24FiuT113A	As pBAD24Fiu with mutation as labelled	This study
pBAD24FiuA117A	As pBAD24Fiu with mutation as labelled	This study
pBAD24FiuI118A	As pBAD24Fiu with mutation as labelled	This study
pBAD24FiuFD124A	As pBAD24Fiu with mutation as labelled	This study
pBAD24FiuS126A	As pBAD24Fiu with mutation as labelled	This study
pBAD24FiuS139A	As pBAD24Fiu with mutation as labelled	This study
pBAD24FiuR142A	As pBAD24Fiu with mutation as labelled	This study
pBAD24FiuY337A	As pBAD24Fiu with mutation as labelled	This study
pBAD24FiuK368A	As pBAD24Fiu with mutation as labelled	This study

References

1. Strong M, Sawaya MR, Wang S, Phillips M, Cascio D, Eisenberg D. Toward the structural genomics of complexes: crystal structure of a PE/PPE protein complex from Mycobacterium tuberculosis. Proceedings of the National Academy of Sciences. 2006;103(21):8060-5.
2. Baba T, Ara T, Hasegawa M, Takai Y, Okumura Y, Baba M, et al. Construction of Escherichia coli K-12 in-frame, single-gene knockout mutants: the Keio collection. Molecular systems biology. 2006;2(1).
